# Gene-based treatment options for Usher type 1C by translational read-through of a nonsense mutation

**DOI:** 10.1186/2046-2530-1-S1-O31

**Published:** 2012-11-16

**Authors:** U Wolfrum, T Goldmann, N Overlack, F Möller, V Belakov, T Baasov, K Nagel-Wolfrum

**Affiliations:** 1 Johannes Gutenberg University of Mainz, Germany; 2 Cell & Matrix Biology, Institute of Zoology, Johannes Gutenberg University of Mainz, Germany; 3 Edith and Joseph Fischer Enzyme Inhibitors Laboratory, Schulich Faculty of Chemistry, Technion-Israel Institute of Technology, Israel

## 

The Usher syndrome (USH) is the most frequent cause of inherited combined deaf-blindness. The ciliopathy is clinically and genetically heterogeneous, assigned to three clinical USH types of which the most severe type is USH1. The *USH1C* gene encodes the PDZ containing scaffold protein harmonin which is expressed in form of numerous alternatively spliced variants. Hamonin binds directly to all USH1/2 proteins and is a key organizer of USH protein networks in photoreceptor cells. So far no effective treatment for the ophthalmic component of USH exists. Translational read-through was introduced as an innovative therapy option for several non-ocular diseases caused by nonsense mutations leading to a premature termination stop. Here we compare the potential of translational read-through inducing drugs (TRIDs), namely PTC124 (currently in clinical phase-II for non-ocular diseases) as well as the designer aminoglycoside NB30 and NB54 as a treatment option for patients carrying a nonsense mutation in the *USH1C* gene causing USH1. We examined read-through in cell culture, retinal cultures and in vivo in murine retinas. Restoration of the harmonin function was tested by GST pull-downs and actin filament bundling. The TRIDs recovered functional harmonin protein and showed an excellent biocompatibility in retinal cultures with read-through vs. toxicity evidently superior for NB54 and PTC124. In vivo administration of NB54 and PTC124 to mice induced recovery of full-length harmonin. The high biocompatibility combined with the sustained read-through efficacies of these novel drugs emphasizes the potential of TRIDs in treating nonsense mutations in USH as well as in other ciliopathies.

